# A case report of Phelan-McDermid syndrome: preliminary results of the treatment with growth hormone therapy

**DOI:** 10.1186/s13052-021-01003-w

**Published:** 2021-03-04

**Authors:** Rui Jin Xie, Tian Xiao Li, Chenyu Sun, Ce Cheng, Jinlin Zhao, Hua Xu, Yueying Liu

**Affiliations:** 1 Affiliated Hospital of Jiangnan University, No. 1000, Hefeng Avenue, Wuxi, 214122 People’s Republic of China; 2 AMITA Health Saint Joseph Hospital Chicago, 2900 N. Lake Shore Drive, Chicago, IL 60657 USA; 3 The University of Arizona College of Medicine at South Campus, 2800 E. Ajo Way, Tucson, AZ 85718 USA

**Keywords:** Phelan-McDermid syndrome, 22q13.3, Treatment, Human growth hormone

## Abstract

**Background:**

Phelan-McDermid syndrome (PMS), also known as 22q13.3 deletion syndrome, is a rare neurodevelopmental syndrome resulting from a deletion of the distal long arm of chromosome 22.

**Case presentation:**

We report a case of a 21 months old Chinese girl presenting with global developmental delay, regression of language skills, unable to understand a few words or walk independently, insomnia, and autism-like behaviors. Copy number variation (CNV) analysis showed a heterozygous loss of SHANK3 gene in the 22q13 region, consistent with a diagnosis of PMS. After treatment with recombinant human growth hormone (rhGH), this patient had an improvement in motor skills and social behaviors. No side effects from rhGH therapy were reported.

**Conclusions:**

This is the first report of using rhGH to treat a Chinese girl diagnosed with PMS. We speculate rhGH could be a reasonable alternative choice for PMS treatment with similar clinical outcomes in comparison to insulin-like growth factor-1(IGF-1). However, further clinical trials are needed to confirm this hypothesis.

**Supplementary Information:**

The online version contains supplementary material available at 10.1186/s13052-021-01003-w.

## Background

Phelan-McDermid syndrome (PMS), also known as 22q13.3 deletion syndrome, is a rare neurodevelopmental syndrome resulting from a deletion of the distal long arm of chromosome 22 [1]. The deletions can vary from 200Kb to 9.2 Mb in size, which leads to a loss of SHANK3 gene [2], which is proved to be the key to cause PMS [3]. Although infants or children with this syndrome manifest diverse clinical features, the most common presentation of PMS include seizure, global developmental delay, hypotonia, absent or severely delayed speech, autism-like spectrum disorder (ASD) [4]. Patients with PMS also present minor dysmorphic facial features, such as large fleshy hands, rounded face, long eyelashes, pointed chin, prominent/dysplastic ears, bulbous nose, full lips, hypoplastic/dysplastic nails, and dolichocephaly [5]. The present recommendation for treatment is insulin-like growth factor-1(IGF-1) [6]. However, this drug has not been approved by China Food and Drug Administration (CFDA, https://www.nmpa.gov.cn/).

Here, we described a patient with a 22q13 deletion (including SHANK3) and a phenotype demonstrating typical clinical features of PMS. The patient was treated by recombinant human growth hormone (rhGH) after obtaining consents from the legal guardians (parents of the patient). To the best of our knowledge, this is the first report of treating PMS by rhGH, with a favorable outcome.

## Case presentation

**Fig. 1:**
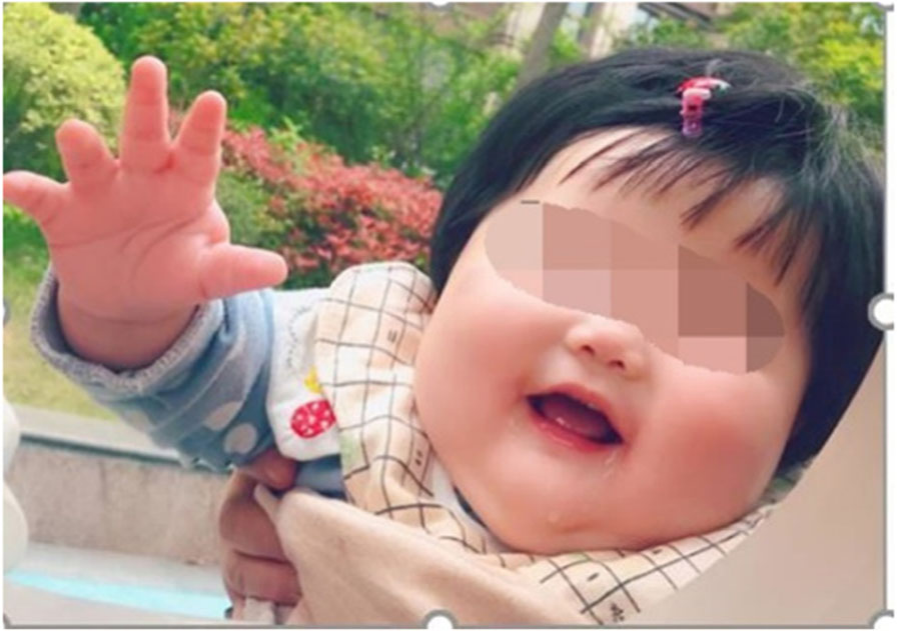
Photograph of patient: rounded face

**Fig. 2:**
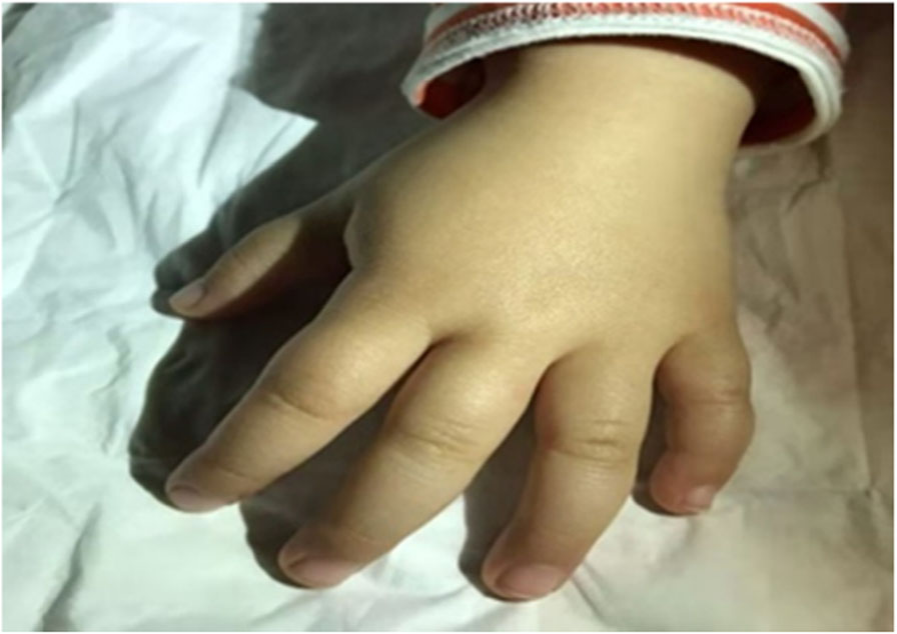
Dysmorphic features of patient: large fleshy hands

A 21 months old Chinese girl was admitted to hospital due to developmental delay after the 14th months. The girl presented with global developmental delay, regression of language skills, unable to understand words or walk independently, and insomnia with difficulty maintaining sleep. She also had autism-like behaviors such as playing alone, abnormal social interactions, poor eye contact and stereotypic behaviors. Relatively large fleshy hands (Fig. 1) and rounded face (Fig. 2) were also noticed. Physical examination showed no other abnormity with normal muscle tension. Her height was 90 cm (+1SD ~ +2SD), weight was 16 kg (> + 3SD), and head circumference was 52 cm (+2SD ~ +3SD).

She was born at 39 weeks of gestation via Caesarean-section, with an Apgar score of 9 and 10 at 1 and 5 min, respectively. Birth weight was 3.45 kg (<+1SD), birth length was 53 cm (+1SD ~ + 2SD), and head circumference was 37 cm (> + 3SD). According to her mother, patient appeared normal before 12th months old without any history of febrile convulsions or other neurological diseases. Also her height and weight were above + 2 standard deviation (SD) during this time, and she was able to sit up at 7th months old and mumble “mama” or “baba” at 8th months old. She was able to ambulate with assistance and understand simple verbal instructions at 12th months old. However, she started to have mild problems to maintain sleep after 12th months old, followed by autism-like behaviors, absent of speech as a regression of language skills, and play in the corner of house. No family history of any mental disorder or genetic illness was identified, and her 11-year-old sister had no symptoms consistent with PMS.

On her 15th months old, electroencephalogram (EEG) and brain magnetic resonance imaging (MRI) were performed without any abnormal findings. Audiometry, pediatric eye exam and echocardiogram were also unremarkable. A bone age of 3 years by skeletal age assessment. The Complete Blood Count (CBC) test, Complete Chemistry Panel (CMP) (including serum creatinine, blood urea nitrogen test, estimated glomerular filtration rate), thyroid function tests urinalysis, myocardial enzyme level (including troponin T, troponin I, creatine kinase, lactate dehydrogenase), serum lactic acid and ammonia were all within normal range. Serum trace metal elements (including copper, zinc, lead and cadmium) were also unremarkable. Serum insulin-like growth factor-1(IGF-1) was 174 ng/ml (normal 55 ~ 327 ng/ml), and insulin-like growth factor binding protein-3 (IGFBP-3) was 5.07 μg /ml (normal 0.7 ~ 3.6 μg/ml). Chinese version of GDS (Gesell Development Scale) was used as neuropsychological development examination, and Infant development was assessed using the development quotient (DQ) according to the following criteria: normal (DQ ≥ 85), borderline (75 ≤ DQ < 85) and abnormal (DQ < 75). The girl had DQ of 58 for “gross motor”, 63 for “fine motor”, 54 for “language”, 57 for “adaptive behavior” and 66 for “personal-social”. The results showed the girl had global developmental delay.

**Fig. 3:**
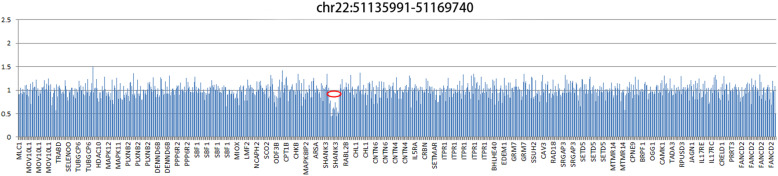
The whole exome sequencing defining the CNV on the patient: the deletion is present in red circle

A course of 5-month physical therapy was initiated. However, no significant clinical progress was observed. Therefore, after obtaining consent from the legal guardians (parents of the patient), genomic DNA was extracted from 2 ml peripheral blood of the patient and sent to the molecular medical Laboratory for whole exome sequencing (Additional file 1: Document S1. The methods of the whole exome sequencing). Firstly, Single Nucleotide Polymorphisms (SNP), Insertion and Deletion (Indel) was analyzed and no suspected variation was detected. Subsequently, copy number variations (CNV) were analyzed based on capture sequencing depth between the patient and normal control. A 33.7Kb-size heterozygous deletion was identified at the region of chr22:51135991–51,169,740 (Fig. 3). The deletion region contained the full length of SHANK3 gene, which previous reported related to Phelan McDermid syndrome in the database of the DECIPHER (https://decipher.sanger.ac.uk/) and OMIM (https://omim.org/). Also, previous reports of heterozygous deletion on the same chr22:51135991–51,169,740 region, which was diagnosed with Phelan McDermid syndrome can be found on the WANFANG Database (http://g.wanfangdata.com.cn/) [7]. In this case, the patient presented with global developmental delay, regression of language skills and autism-like behaviors, met the diagnostic criteria of Phelan McDermid syndrome. Combined with the clinical symptoms and genetic result, the patient can be diagnosed as Phelan McDermid syndrome.

As IGF-1 therapy has not been approved by CFDA for PMS, a consent was obtained from the parents of this patient, after extensive discussion regarding the potential benefits versus risks of subcutaneous rhGH. rhGH treatment was started at 0.075 IU/kg (ChangchunJinsaiGenSci Co., Ltd., GH30IU) once daily by subcutaneous injection for 3 months.

No severe or common adverse events were observed throughout the treatment course, Post- treatment serum IGF-1 was 258 ng/ml, and IGFBP-3 was 5.4 μg /ml. Another neuropsychological development examination was repeated according to the Chinese version of GDS. This patient had DQ of 84 for “gross motor”, 84 for “fine motor”, 78 for “language”, 72 for “adaptive behavior” and 73 for “personal-social”. These results indicated significant improvements in serum IGF-1 and IGFBP-3, and motor skills. Clinically, this patient was also able to communicate with parents by simple one sentence like wants watch cartoon and play outside, get along well with other children, walk up and down two or three stairs independently.

## Discussion and conclusions

We reported this case with typical clinical presentations and genetic findings of PMS in a 21 months old Chinese girl. She had global developmental delay, regression of language skills, unable to understand words and walk independently, difficulty maintaining sleep, autism-like behaviors, large fleshy hands and rounded face. This girl also showed a poorer gross motor development than fine motor development according to the first Chinese version of GDS on 15th months. This unusual phenomenon can be seen on the developmental delay children at 12 and at 17 [8], and we assumed it might relate to her hypotonic muscle. Copy number variation analysis of the patient revealed a heterozygous 33.7Kb deletion of chr22:51135991–51,169,740 region encompassing the whole SHANK3 gene, which is considered to be the cause of the neurobehavioral symptoms. PMS is a rare disease neurodevelopmental syndrome, with only about 1600 patients reported worldwide [2]. IGF-1 was proved to reverse neuron synaptic deficits in PMS patients by causing loss of SHANK3-containing synapses and gain of PSD95-containing synapses [9]. The first clinical trial of IGF-1 in children with PMS [6, 10] included 9 children between 5 and 15 years old with PMS who had confirmed SHANK3 deletions or mutations. Trial followed a randomized, placebo-controlled, cross over format with 12 weeks in each treatment arm (IGF-1 and placebo), separated by a 4-week wash-out phase [6]. IGF-1 was initiated at 0.04 mg/kg twice daily by subcutaneous injection, and increased every week as tolerated, by 0.04 mg/kg per dose to a maximum of 0.12 mg/kg twice daily [6]. It resulted in a clinical improvement in social impairments and restrictive behaviors, measured by changes in the Aberrant Behavior Checklist Social Withdrawal subscale and the Restricted Behavior Subscale of the Repetitive Behavior Scale [8, 11].

Base on this literature, we speculated rhGH might be an alternative choice, which was hypothesized in this case to improve symptoms of PMS by increasing serum IGF-1, IGFBP-3 and acid-labile subunit (ALS) level. As this is the first reported case of using rhGH to treat PMS, we used the minimum dosage of RhGH therapy 0.075 IU/kg to ensure its safety according to Chinese official guideline for using RhGH [12]. Growth hormone (GH) and IGF-1 are both growth factors exerting trophic effects on neuronal regeneration in the central nervous system (CNS) and peripheral nervous system (PNS). They are both determinant regulators of cellular function, and an impaired release of GH and IGF-1 with advancing age leads to severe alterations in tissue structures and functions, especially within the brain [13]. GH is secreted by the anterior pituitary gland and GH is the major stimulator of IGF-1 secretion from the liver. Also, the production of IGFBP-1 and -2 is inhibited by GH whereas production of IGFBP-3, − 4 and − 5 is stimulated by GH. IGF-1 is mainly bound to IGFBP-3 and this binary complex then binds to a large protein called ALS, thus a deficiency of ALS in children is associated with growth retardation [14].

Compare with IGF-1, rhGH is of low cost and more accessible, which also improved the outcome and symptoms of PMS patient reported in our case, including motor skills and autism-like behaviors. Our case might provide a new therapeutic approach for PMS. However, because this is only one case with a short follow-up period, further studies and clinical trials are still warranted to evaluate rhGH in treating PMS.

## Supplementary Information

**Additional file 1.**

## Data Availability

Not applicable.

## Abbreviations

PMS: Phelan-McDermid syndrome
rhGH: Recombinant human growth hormone
IGF-1: Insulin-like growth factor-1
GH: Growth hormone
IGFBP-3: Insulin-like growth factor binding protein-3
ALS: Acid-labile subunit
CNV: Copy number variation
GDS: Gesell Development Scale
DQ: Development quotient
