# Correction to: A case report of Phelan-McDermid syndrome: preliminary results of the treatment with growth hormone therapy

**DOI:** 10.1186/s13052-021-01038-z

**Published:** 2021-04-14

**Authors:** Rui Jin Xie, Tian Xiao Li, Chenyu Sun, Ce Cheng, Jinlin Zhao, Hua Xu, Yueying Liu

**Affiliations:** 1 Affiliated Hospital of Jiangnan University, No. 1000, Hefeng Avenue, Wuxi, 214122 People’s Republic of China; 2 AMITA Health Saint Joseph Hospital Chicago, 2900 N. Lake Shore Drive, Chicago, IL 60657 USA; 3 The University of Arizona College of Medicine at South Campus, 2800 E. Ajo Way, Tucson, AZ 85718 USA

**Correction to: Ital J Pediatr 47, 49 (2021)**

**https://doi.org/10.1186/s13052-021-01003-w**

Figure 1 was replaced with a more appropriate image due to privacy issues in the original article [1]. All authors agreed to this correction.

**Fig. 1:**
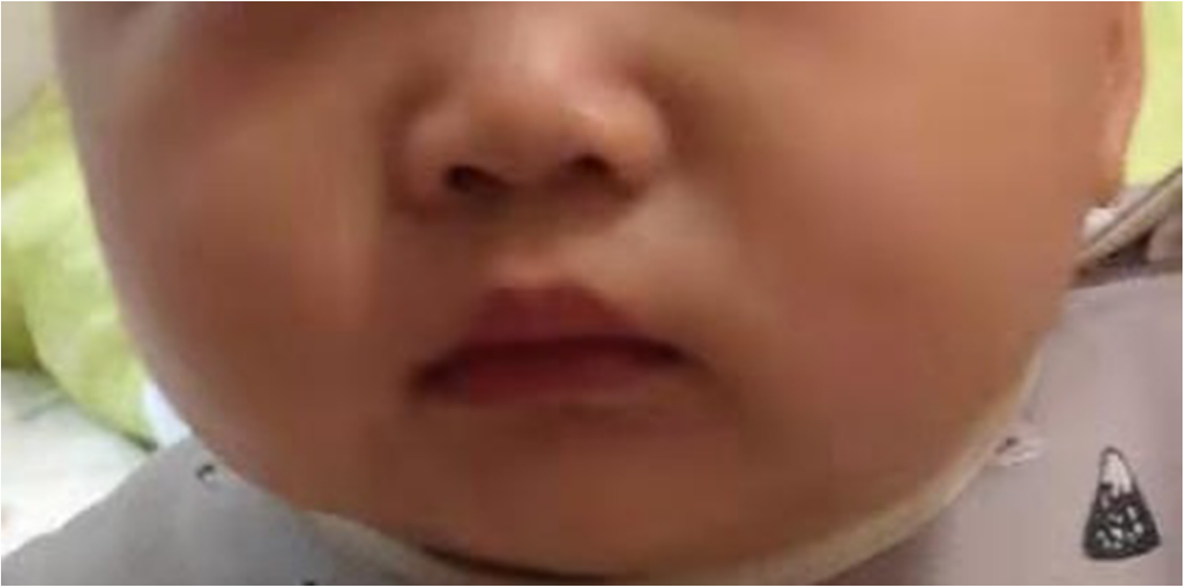
Photograph of patient: rounded face
