# Supplementary material for: A case report of Phelan-McDermid syndrome: preliminary results of the treatment with growth hormone therapy
Source: Ital J Pediatr. 2021 Mar 4;47:49. doi: 10.1186/s13052-021-01003-w (PMC7934562; doi:10.1186/s13052-021-01003-w)
Supplement: Supplementary file 1 — Additional file 1. [file 13052_2021_1003_MOESM1_ESM.docx]

Whole exome sequencing

Genomic DNA was extracted from the peripheral blood by using QIAamp DNA Mini Kit (QIAGEN). DNA was quantified with Nanodrop 2000 (Thermal Fisher Scientific, DE). A minimum of 3 ug DNA was used for the indexed Illumina libraries according to the manufacturer’s protocol. The DNA fragments with sizes ranging from 350 bp to 450 bp and those including the adapter sequences were selected for DNA libraries. The constructed libraries were captured by xGen Exome Research Panel v2 (IDT, Coralville, Iowa, USA). The enriched libraries were sequenced on an Illumina HiSeq XTen sequencer (Illumina, San Diego, CA, USA) for paired-end reads of 150 bp. 100~200X mean coverage was chosen and 12Gb are sequenced per patient. Following sequencing, the raw image files were processed by using Bcl2Fastq software (Bcl2Fastq 2.18.0.12, Illumina, Inc.) for base calling and raw data generation. Low-quality variations were filtered out by a quality score of ≥20. SOAP aligner software (SOAP2.21; soap.genomics.org.cn/soapsnp.html) was then used to align the clean reads with the reference human genome (GRCh37). Polymerase chain reaction (PCR) duplicates were removed by using the Picard program. Subsequently, single nucleotide polymorphisms (SNPs) were determined by using the SOAPsnp program, reads were realigned by using Burrows-Wheeler Aligner software 0.7.15, and the insertions and deletions (InDels) were detected by using Genome Analysis Toolkit (GATK) software 3.7. Meanwhile, copy number variations were analyzed based on the depth of per exon among patient and normal controls. The ratio lower than 0.75 indicated deletion, while a ratio higher than 1.25 indicated duplication.
